# Personalized Estimates of Brain Cortical Structural Similarity in Major Depressive Disorder: Evidence from a Multi-Site Neuroimaging Dataset

**DOI:** 10.3390/diagnostics16111632

**Published:** 2026-05-26

**Authors:** Xuetian Sun, Yuhao Shen, Jiajia Zhu, Yongqiang Yu

**Affiliations:** 1 Department of Radiology, The First Affiliated Hospital of Anhui Medical University, Hefei 230022, China; 2346010218@stu.ahmu.edu.cn (X.S.); 2346010217@stu.ahmu.edu.cn (Y.S.); 2 Research Center of Clinical Medical Imaging, Hefei 230032, China; 3 Anhui Provincial Key Laboratory for Brain Bank Construction and Resource Utilization, Hefei 230032, China

**Keywords:** major depressive disorder, person-based similarity index, magnetic resonance imaging, cortical morphology, REST-meta-MDD

## Abstract

**Background**: Major depressive disorder (MDD) is increasingly recognized as a highly heterogeneous disorder. Although the person-based similarity index (PBSI) provides a useful framework for characterizing individualized brain structural similarity, existing studies in MDD remain limited by either small samples or a lack of integration across different morphological features. **Methods**: We used structural MRI data from 1442 patients with MDD and 1277 healthy controls to calculate PBSI scores of cortical morphology measures based on cortical thickness (CT), cortical volume (CV), cortical surface area (SA), and sulcal depth (SD). Group comparisons of whole-brain PBSI and regional contributions to PBSI scores were then performed, and a subgroup analysis in 243 first-episode, drug-naive (FEDN) patients with MDD was further conducted. **Results**: Patients with MDD showed significant alterations in PBSI. Specifically, PBSI scores were significantly reduced for CT, CV, and SD, whereas no significant group difference was observed for SA in the main analysis. Analyses of regional contributions to PBSI further revealed significant between-group differences across multiple cortical regions. These alterations were mainly distributed in the default mode, ventral attention, and visual networks for CT; in the default mode, ventral attention, sensorimotor, and visual networks for CV; and in the default mode, dorsal attention, frontoparietal, and sensorimotor networks for SD. Similar patterns were also observed in the FEDN MDD subgroup. **Conclusions**: These findings provide neurobiological evidence for the marked structural heterogeneity of MDD and highlight the potential of PBSI as an individualized neuroimaging marker for more precise diagnosis and personalized intervention.

## 1. Introduction

Major depressive disorder (MDD) is a prevalent and disabling psychiatric disorder that imposes substantial personal, societal, and economic burdens, and its marked clinical heterogeneity remains a major barrier to precise diagnosis and individualized treatment [1,2]. Although substantial efforts have been made in the past decades, the etiology of MDD remains elusive. Advancements in neuroimaging techniques, and magnetic resonance imaging (MRI) in particular, have increasingly enabled the in vivo investigation of brain structure and function with high precision, making it more feasible to explore the neuropathological underpinnings of MDD. Numerous case–control neuroimaging studies have consistently identified significant cortical morphological alterations in patients with MDD, particularly in key regions involved in affective and cognitive processing, including the prefrontal cortex, anterior cingulate cortex, and temporal cortex [3,4,5]. However, group-level analytical approaches may obscure substantial inter-individual variability, which limits reproducibility and impedes clinical translation.

With the emergence of precision psychiatry, neuroimaging research has increasingly moved beyond group-average comparisons toward individual-level characterization of brain abnormalities [6,7]. Although normative modeling has provided an important framework for quantifying subject-specific deviations from healthy reference distributions [8,9,10,11], approaches that focus solely on deviation from a normative range may not fully capture the degree to which an individual resembles or diverges from others within the same diagnostic group. In this context, the person-based similarity index (PBSI) has been proposed as a complementary individualized metric for quantifying the similarity of an individual’s brain structural profile to that of other members of the same group [12]. Specifically, PBSI integrates whole-brain multiregional morphometric measures into an individual-specific feature vector and quantifies the overall similarity between one individual and other group members using Spearman rank correlations between these feature vectors. This metric reflects the extent to which an individual’s brain structural phenotype is typical or distinctive relative to the group pattern and has been shown to be biologically meaningful, with evidence of heritability, robustness to imaging parameters, and associations with cognitive function [12,13]. PBSI has been applied to several psychiatric and neurological disorders, including schizophrenia [14,15,16,17], bipolar disorder [13], multiple sclerosis [18], and autism spectrum disorder [19], with studies consistently reporting lower PBSI scores in patient groups than in healthy controls. These findings suggest that greater inter-individual variability in brain structure may represent a common feature across brain disorders.

Importantly, although whole-brain PBSI summarizes the similarity between an individual’s overall brain morphological profile and that of the group, it does not identify the specific regions contributing to this similarity or heterogeneity. Because PBSI is determined jointly by morphometric measurements across all cortical regions, evaluating regional contributions is important for understanding the biological basis of inter-individual variation in brain structure. Using the leave-one-region-out (LORO) strategy proposed by Doucet et al. [13], regional contributions can be assessed by systematically excluding each region and recalculating PBSI. This approach enables the identification of regions that contribute to within-group similarity or heterogeneity, thereby providing more precise localization of disease-related structural variation.

The application of PBSI in MDD remains limited. Existing studies have largely focused on single morphometric features [20,21] (e.g., gray matter volume), and individualized structural similarity across multiple cortical morphological measures has not been systematically evaluated. This limitation is important because different cortical features may reflect partly distinct neurobiological processes [22]. Specifically, cortical thickness (CT) has been linked to neuronal density and laminar organization, cortical surface area (SA) is mainly influenced by neurogenesis and radial glial proliferation, cortical volume (CV) integrates information from both thickness and surface area, and sulcal depth (SD) reflects cortical folding and early neurodevelopmental processes [23,24,25,26,27,28]. A multi-feature cortical PBSI framework incorporating these measures may therefore provide a more comprehensive characterization of individual variation in brain structure in MDD. In addition, the clinical heterogeneity of MDD is reflected not only in symptom profiles, but also in differences in illness course and treatment response. First-episode, drug-naive (FEDN) MDD represents an early stage of the disorder and is less affected by recurrent episodes, chronicity, and medication exposure, providing a valuable window for investigating primary neurobiological alterations in MDD [29]. Studies have shown that structural abnormalities are already present at illness onset in FEDN MDD [30,31,32], including gray matter volume alterations in the thalamus, hippocampus, and prefrontal cortex, as well as disrupted structural covariance network topology [33,34]. However, individual-level brain structural similarity in FEDN MDD remains unclear. Investigating PBSI in this subgroup may help identify early structural phenotypes and evaluate the robustness of findings in the broader MDD population.

Our objective in this exploratory study was to investigate alterations in PBSI in patients with MDD. To achieve this goal, we constructed four cortical PBSI measures based on different surface-based morphometric metrics: CT, CV, SA and SD, based on the largest sMRI data from 1442 MDD patients and 1277 healthy controls collected by the Depression Imaging REsearch ConsorTium (DIRECT) in China [35]. Subsequently, group comparisons of PBSI were performed between patients with MDD and healthy controls. In addition, a LORO analysis was conducted to evaluate the contribution of individual cortical regions to group-level similarity or heterogeneity. Finally, to minimize the potential confounding effects of illness duration and medication exposure, a subgroup analysis was performed in a cohort of FEDN MDD patients using the same analytical procedure.

## 2. Materials and Methods

### 2.1. Participants

A total of 1660 MDD patients and 1341 healthy controls were recruited by twenty-three research groups from the DIRECT consortium [35]. The studies were approved by local Institutional Review Boards, and written informed consent was obtained from all participants. All ethical regulations relevant to human research participants were followed. Participants were excluded if they had no information on age or sex. Both groups had demographic data, including age, sex, and education. For MDD patients, clinical characteristics included onset age, illness duration, first-episode or recurrent status, episode number, medication use, 17-item Hamilton Rating Scale for Depression (HAMD), and Hamilton Rating Scale for Anxiety (HAMA).

### 2.2. Image Quality Control

Image quality control was performed by a well-trained researcher (X.S.) with prior expertise in neuroimaging data inspection. Before the formal quality control procedure, this researcher had demonstrated high inter-rater agreement with a second expert rater on a pilot subset of 100 participants’ structural MRI scans (Cohen’s κ > 0.85). Although automated quality control strategies, such as the Euler number, provide time-efficient and reproducible alternatives, manual visual inspection remains important for detecting visible artifacts, organic brain abnormality, and segmentation inaccuracies that may not be fully captured by automated indices [36]. Therefore, we adopted two manual quality control strategies for the structural MRI images: (1) visual inspection with exclusion of visible artifacts, organic brain abnormality, and inaccurate segmentation, and (2) visual inspection with manual editing, where the inaccurate segmentation was manually edited. Furthermore, to account for potential confounding effects of development and aging, participants younger than 18 or older than 65 were excluded from the study. The final sample consisted of 1442 MDD patients and 1277 healthy controls from 22 sites. Demographic and clinical characteristics of the samples are shown in Supplementary Table S1. With the participants pooled together, we found that the patient group had a higher proportion of females, was older, and had a lower level of education relative to controls (see Supplementary Table S2 for detailed information). Therefore, we regressed the effects of age, sex, and years of education in subsequent group comparison analysis.

### 2.3. Structural MRI Data Processing

The processing of structural MRI data was conducted using the toolbox for Data Processing & Analysis for Brain Imaging on Surface (DPABISurf) [37], which is based on FreeSurfer [38], ANTs [39], FSL [40], SPM [41], PALM [42], dcm2niix [43], GNU Parallel [44], MATLAB R2022b (The MathWorks Inc., Natick, MA, USA), and Docker (https://docker.com). The processing pipeline for T1-weighted data included the following steps: (i) converting the data to BIDS format [45]; (ii) correcting intensity nonuniformity in the T1-weighted image using N4BiasFieldCorrection [46]; (iii) performing skull stripping with ANTs [47]; (iv) reconstructing cortical surface models (gray-white boundary and pial surfaces) using the recon-all command in FreeSurfer 6.0.1 [38]; (v) registering the individual’s cortical surface nonlinearly to the FreeSurfer fsaverage surface space; (vi) calculating 4 types of surface-based morphological metrics, including CT, CV, SA, and SD; and (vii) smoothing the data using the mri_surf2surf command of FreeSurfer with a Gaussian kernel of 15 mm full width at half maximum (FWHM) for CT, CV and SA, and 25 mm FWHM for SD. The larger smoothing kernel was applied to SD due to the underlying nature of this folding metric that reflects contributions from both sulci and gyri, such that the smoothing kernel size should exceed the distance between a gyral crown and a sulcal fundus.

### 2.4. Computation of the PBSI Scores

**Figure 1:**
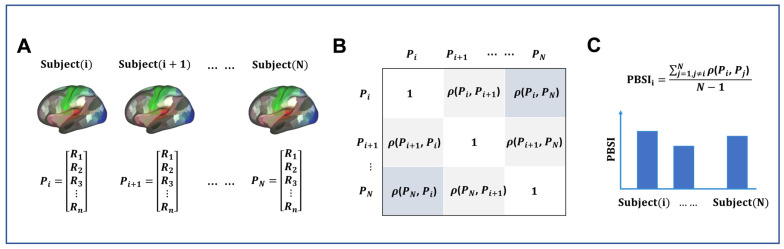
Pipeline for computing the person-based similarity index. The Person-Based Similarity Index (PBSI) quantifies the similarity of an individual’s morphometric profile to those of all other individuals in the same group. (**A**) For each subject, the regional structural measures (R) are concatenated into a single vector (P), which represents that subject’s structural profile for the given measure set. (**B**) Spearman’s correlation ρ is computed between every pair of individual profiles. (**C**) The PBSI of subject(i) is the average of all pairwise correlations between subject(i) and all other subjects within the same group.

In this study, PBSI was calculated at the individual level in both the MDD and HC groups following previously established frameworks [12,14,18,19,48,49,50,51]. Four cortical surface morphological measures were examined, including CT, CV, SA, and SD. The cortex was parcellated using the Human Connectome Project multimodal parcellation (HCP-MMP) atlas [52], which divides each hemisphere into 180 regions, yielding 360 cortical regions in total. For each morphological measure, morphological values from all vertices within each cortical region were extracted to generate an individual brain structural feature vector, defined as P = (R1, R2, …, R360) (Figure 1A). Given the potential bias arising from site effects in multi-site neuroimaging data, ComBat harmonization [53] was applied to reduce non-biological variability. In this study, group, age, sex, and education were included as covariates in the ComBat model to preserve important biological trends in the data and avoid overcorrection. Within each group, Spearman correlation coefficients were then calculated between each participant’s structural feature vector and those of all other participants for the same measure (Figure 1B). For each participant, this step yielded *N* − 1 correlation coefficients, where *N* denotes the number of individuals in the corresponding group. The mean of these coefficients was defined as the PBSI score, reflecting the average similarity between that participant and all other group members (Figure 1C). The MATLAB function used to compute the PBSI score is available at: https://www.mathworks.com/matlabcentral/fileexchange/69158-similarityscore (accessed on 5 January 2025). PBSI values range from −1 to 1, consistent with Spearman correlation coefficients, although they are typically positive in large-scale healthy and clinical datasets [14,18,19,48,49,50]. Higher PBSI values indicate greater similarity between an individual’s cortical morphometric profile and those of other individuals within the same diagnostic group. In contrast, lower PBSI values reflect reduced within-group structural similarity and greater inter-individual heterogeneity. This procedure was performed independently for CT, CV, SA, and SD, yielding four PBSI metrics for each participant: PBSI-CT, PBSI-CV, PBSI-SA, and PBSI-SD.

### 2.5. Contribution of Regional Brain Measures to the PBSI

Regional contributions to PBSI were quantified using a LORO approach, performed separately for each diagnostic group (MDD and HC) and each morphometric metric (CT, CV, SA, and SD). For each participant, the PBSI score was recalculated iteratively after excluding one brain region at a time. The contribution of region i was defined as the difference between the original PBSI score and the recalculated PBSI score obtained after excluding region i. A positive value indicates that the region increased the individual’s similarity to the group-level pattern, whereas a negative value indicates that the region reduced it. Values close to zero suggest a negligible contribution to the overall similarity estimate.

### 2.6. Statistical Analysis

#### 2.6.1. Between-Group Comparisons of PBSI Scores

For each morphometric metric (CT, CV, SA, and SD), group differences in PBSI scores between the MDD and HC groups were tested using a general linear model (GLM), with age, sex, and years of education included as covariates, following previous studies [18,54]. Statistical significance was set at *p* < 0.05.

#### 2.6.2. Between-Group Comparisons of Regional Contributions to PBSI

Group differences in regional contributions to PBSI were examined for each cortical region using a GLM with age, sex, and years of education as covariates. To control for multiple comparisons across the 360 ROIs, Bonferroni correction was applied, and a corrected threshold of *p* < 0.05/360 was considered statistically significant. Regions showing significant between-group differences were further mapped onto the Yeo 7-network parcellation [55] to examine the network-level distribution of these effects.

### 2.7. Subgroup Analysis

To minimize the potential effects of illness duration and medication exposure, a subgroup analysis was conducted in patients with FEDN MDD. FEDN MDD patients were defined as individuals who were experiencing their first depressive episode, had an illness duration of less than one year, and had not received any treatment with antidepressant medications at the time of assessment. A total of 243 FEDN MDD patients were included in the subgroup analysis, with detailed demographic and clinical information provided in Supplementary Table S3. Compared with healthy controls, FEDN MDD patients showed no significant differences in age or sex, but exhibited a significant difference in education (Supplementary Table S4). The same image processing, PBSI calculation, regional contribution analysis, and group comparison procedures used in the main analysis were subsequently applied to the FEDN MDD subgroup.

## 3. Results

### 3.1. Group Differences in PBSI

#### 3.1.1. MDD Patients Versus Healthy Controls

**Figure 2:**
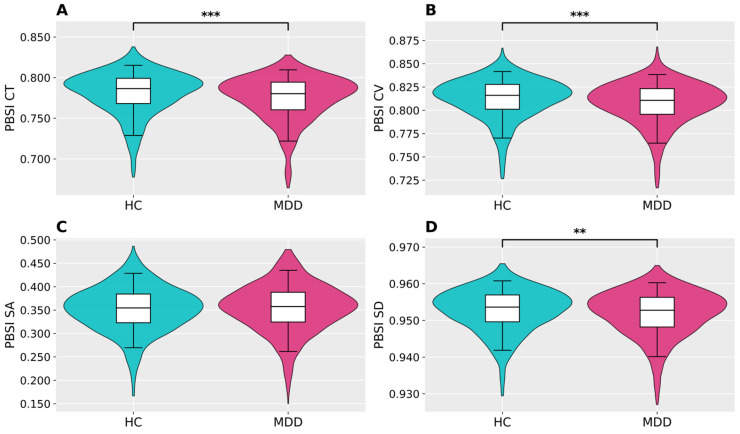
(**A**–**D**) Group differences in PBSI scores between MDD patients and healthy controls for four cortical morphological measures. Symbol meanings: ** *p* < 0.01, *** *p* < 0.001. Abbreviations: CT, cortical thickness; CV, cortical volume; HC, healthy control; MDD, major depressive disorder; PBSI, person-based similarity index; SA, surface area; SD, sulcal depth.

Group comparisons revealed that, relative to healthy controls, patients with MDD exhibited significantly lower PBSI scores across multiple cortical morphometric measures, as illustrated in Figure 2 and Supplementary Table S5. Given that PBSI was calculated within each diagnostic group, these reductions indicate reduced within-group structural similarity and greater inter-individual heterogeneity in cortical morphometric profiles among patients with MDD. Specifically, patients with MDD showed significantly lower PBSI-CT and PBSI-CV scores than healthy controls (*p* < 0.001). Similarly, PBSI-SD was also significantly reduced in the MDD group (*p* = 0.002). In contrast, no significant group difference was observed for PBSI-SA (*p* = 0.176).

#### 3.1.2. FEDN MDD Patients Versus Healthy Controls

**Figure 3:**
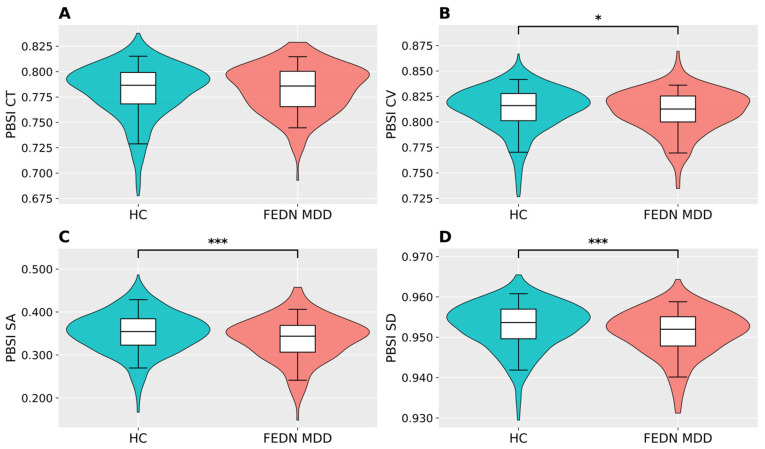
(**A**–**D**) Group differences in PBSI scores between FEDN MDD patients and healthy controls for four cortical morphological measures. Symbol meanings: * *p* < 0.05, *** *p* < 0.001. Abbreviations: CT, cortical thickness; CV, cortical volume; FEDN MDD, first-episode drug-naive major depressive disorder; HC, healthy control; PBSI, person-based similarity index; SA, surface area; SD, sulcal depth.

Group comparisons in the FEDN subgroup also revealed significant differences in PBSI between FEDN MDD patients and healthy controls (Figure 3 and Supplementary Table S6). With respect to PBSI-CV, FEDN MDD patients showed significantly reduced PBSI scores compared with healthy controls (*p* = 0.012), consistent with the main analysis. In addition, PBSI-SA and PBSI-SD were also significantly lower in FEDN MDD patients (*p* < 0.001). By contrast, no significant group difference was observed for PBSI-CT (*p* = 0.788). Overall, the FEDN subgroup exhibited a pattern of PBSI alteration that was broadly consistent with the main analysis, while also showing metric-specific differences.

### 3.2. Regional Contributions to the PBSI

**Figure 4:**
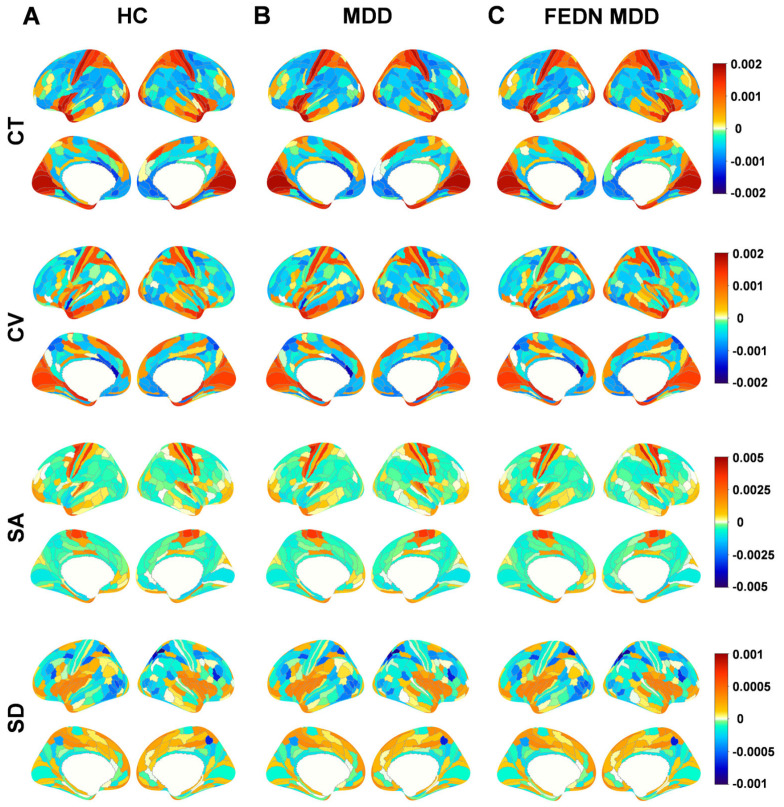
(**A**–**C**) Distribution maps of average regional contributions to the PBSI score in each group. Warm colors indicate brain regions with positive contributions to the PBSI score, whereas cool colors indicate brain regions with negative contributions. The color bar indicates the range of contribution values. Abbreviations: CT, cortical thickness; CV, cortical volume; FEDN MDD, first-episode drug-naive major depressive disorder; HC, healthy control; MDD, major depressive disorder; PBSI, person-based similarity index; SA, surface area; SD, sulcal depth.

Regional contribution maps of PBSI for CT, CV, SA, and SD are shown in Figure 4 for healthy controls (Figure 4A), patients with MDD (Figure 4B), and FEDN MDD patients (Figure 4C). Across all three groups, the spatial distribution of regional contributions varied across morphometric measures, indicating that different cortical features contributed differently to whole-brain PBSI.

### 3.3. Group Differences in Regional Contributions to PBSI

#### 3.3.1. MDD Patients Versus Healthy Controls

**Figure 5:**
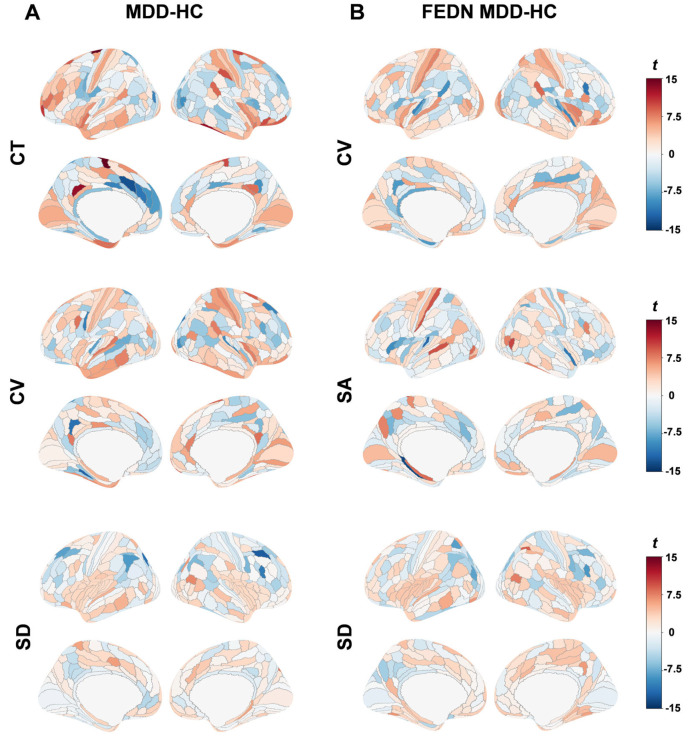
(**A**,**B**) Distribution maps of group differences in regional contributions to PBSI score. Abbreviations: CT, cortical thickness; CV, cortical volume; FEDN MDD, first-episode drug-naive major depressive disorder; HC, healthy control; MDD, major depressive disorder; PBSI, person-based similarity index; SA, surface area; SD, sulcal depth.

Significant between-group differences in regional contributions to PBSI were observed between the MDD and HC groups for three cortical morphometric measures, including CT, CV, and SD (Figure 5A and Supplementary File S1). Specifically, based on regional contributions to the PBSI-CT score, 146 cortical regions showed significant group differences between MDD patients and healthy controls (*p* < 0.05, Bonferroni corrected). Mapping these regions onto the Yeo 7-network parcellation showed that they were mainly distributed in the default mode network (DMN), ventral attention network (VAN), and visual network (VN) (Supplementary Figure S1A). For PBSI-CV, 129 cortical regions showed significant between-group differences (*p* < 0.05, Bonferroni corrected). These regions were primarily distributed in the DMN, VAN, sensorimotor network (SMN), and VN (Supplementary Figure S1A). For PBSI-SD, 47 cortical regions showed significant between-group differences (*p* < 0.05, Bonferroni corrected). These regions were also mainly distributed in the DMN, dorsal attention network (DAN), frontoparietal network (FPN), and SMN (Supplementary Figure S1A).

#### 3.3.2. FEDN MDD Patients Versus Healthy Controls

Significant between-group differences in regional contributions to PBSI were also observed between the FEDN MDD and HC groups for three cortical morphometric measures, including CV, SA, and SD (Figure 5B and Supplementary File S1). Specifically, 118 cortical regions showed significant group differences based on regional contributions to the PBSI-CV score (*p* < 0.05, Bonferroni corrected). Mapping these regions onto the Yeo 7-network parcellation showed that they were mainly distributed in the SMN, VAN, and DMN (Supplementary Figure S1B). For PBSI-SA, 75 cortical regions showed significant between-group differences, primarily distributed in the DMN, SMN, and VN (Supplementary Figure S1B). For PBSI-SD, 88 cortical regions showed significant between-group differences, which were mainly distributed in the VN, SMN, and VAN (Supplementary Figure S1B). Overall, the FEDN subgroup showed a pattern of altered regional contributions to PBSI that was partly consistent with the main analysis, while also exhibiting metric-specific differences in the number and network distribution of significant regions.

## 4. Discussion

### 4.1. Principal Findings

The present study represents a large-scale, multi-site investigation of individual-level structural similarity in MDD using the PBSI framework across four cortical morphometric measures (CT, CV, SA, and SD). Group comparisons revealed widespread reductions in PBSI in patients with MDD across multiple morphometric measures, including CT, CV, and SD. These findings indicate reduced within-group structural similarity and greater inter-individual heterogeneity in cortical morphometric profiles among patients with MDD. From a clinical perspective, this pattern is consistent with the marked heterogeneity of MDD, including variability in symptom presentation, illness course, and treatment response. Moreover, significant differences were observed in the regional contributions to PBSI across numerous cortical regions, with distinct morphometric-specific patterns of alteration. Among the cortical regions showing significant group differences in regional contributions to PBSI, those identified based on CT were primarily located within the DMN, VAN, and VN, whereas those identified based on CV were more widely distributed across the DMN, VAN, SMN, and VN, and those based on SD were distributed across the DMN, DAN, FPN, and SMN. Similar patterns were also observed in the FEDN MDD subgroup, suggesting that these alterations in individual-level structural similarity and regional contributions to PBSI may already be present at the early stage of the disorder. Collectively, these findings suggest that PBSI is capable of capturing alterations in individual-level brain structural similarity across multiple morphometric measures in MDD, and may provide a novel perspective for understanding the marked heterogeneity of this disorder.

### 4.2. PBSI Alterations Across Cortical Morphometric Measures

We found that PBSI scores for CT, CV, and SD were significantly lower in patients with MDD than in healthy controls, indicating that brain structural patterns in MDD deviate more markedly from the normative profile of healthy controls and reflecting a process of structural “de-typicalization.” This finding accords with the growing recognition that heterogeneity is a central feature of psychiatric disorders [51,56]. Brain structural abnormalities in MDD are often highly individualized, and such marked inter-individual variability underscores a major limitation of conventional group-average approaches, which may obscure biologically meaningful patient-specific alterations [57,58]. This interpretation is further supported by the FEDN MDD subgroup, in which significant reductions in PBSI were observed for CV, SA, and SD even after minimizing the potential influence of medication exposure and illness chronicity. Together, these findings suggest that PBSI abnormalities may emerge early in the course of MDD and may reflect inherent neuroanatomical features of the disorder [30,59,60], rather than being attributable to illness duration or medication effects. In this context, PBSI provides a useful framework for quantifying such individualized structural variation, thereby enhancing its potential for clinical translation. Accordingly, PBSI may not only serve as a potentially informative neuroimaging marker for MDD, but may also have potential utility in predicting treatment response and guiding personalized intervention strategies.

The differential group effects observed across cortical morphometric measures may reflect distinct underlying biological mechanisms. Reduced similarity in CT may indicate abnormalities in synaptic pruning or dendritic atrophy associated with chronic stress [54,61]. Consistent with this interpretation, previous studies have shown that CT alterations are more prominent in adult depression and may be more strongly influenced by environmental factors [3,4]. Reduced similarity in CV may reflect cumulative neuroanatomical burden [62], as recurrent depressive episodes have been associated with neuronal shrinkage and glial loss in prefrontal and limbic regions, although the spatial distribution of these changes appears to vary considerably across individuals [63]. Reduced similarity in SD, on the other hand, may suggest a neurodevelopmental contribution to morphological abnormalities in MDD [64,65], given the close relationship of sulcal depth with white matter tension and early cortical folding [66,67]. By contrast, SA did not show significant alterations in the main analysis. As cortical surface area is known to be highly heritable and largely established during early neurodevelopment [68], this finding may suggest that structural heterogeneity in MDD is more strongly related to acquired pathological remodeling than to purely genetic influences [69].

### 4.3. Regional Contributions to PBSI Alterations

When interpreting these regional findings, it should be emphasized that the LORO analysis provides data-driven evidence for regional contributions to group-level PBSI differences, rather than direct evidence of region-specific pathophysiological mechanisms. Thus, the cortical regions identified in this analysis should be understood as regions contributing to altered individual-level structural similarity in MDD. The assignment of these regions to large-scale functional networks was used only as a descriptive framework to summarize the spatial distribution of the LORO results. The present regional findings should therefore be regarded as exploratory and hypothesis-generating. Previous neuroimaging studies provide useful context for discussing the potential relevance of these regions.

In the between-group comparison of regional contributions to PBSI between patients with MDD and healthy controls, significant differences were observed in multiple regions within the DMN and VAN based on CT and CV. Within the DMN, the contributions of the left d32, 9m and the right 31pd were increased, whereas those of the left a10p, 31p and the right 47s were decreased. As these regions are broadly involved in self-referential processing, internal cognition, and emotion-related information integration, their cortical “de-typicalization” may be related to core depressive symptoms such as rumination [52,70,71]. In addition, the left p32pr and p24pr within the VAN showed increased contributions based on CT. Given the central role of the anterior cingulate and adjacent paralimbic regions in salience processing and inter-network coordination, structural alterations in these regions may contribute to reduced emotion regulation efficiency and impaired cognitive flexibility in MDD [72,73,74]. Based on the SD and CV analyses, regions showing significant between-group differences in contribution were mainly located in the FPN and DAN, including the right 8Av and IFJa and the left IFJp and IP2 within the FPN, as well as the left 7AL and IPS1 and the right IP0 and TPOJ3 within the DAN. These findings suggest that structural heterogeneity in MDD extends beyond the DMN and VAN to regions involved in executive control, attentional allocation, and cognitive flexibility. Notably, the altered contribution of IFJa indicates that prefrontal regions supporting task switching and cognitive control may already show deviations in cortical folding, potentially reflecting the combined effects of early cortical development and subsequent pathological remodeling [72,75,76]. Beyond higher-order networks, abnormalities were also evident in lower-order networks. Specifically, significant regional contribution differences were identified in the bilateral 6mp of the SMN based on CT, the left PHA2 of the VN based on CV, and the right V6A of the VN based on SD. These findings indicate that structural heterogeneity in MDD extends beyond higher-order cognitive systems to sensorimotor and visual networks, supporting a cross-network distribution pattern [55,77]. Structural “de-typicalization” in SMN- and VN-related regions may be associated with psychomotor retardation [78,79,80], somatic pain [81,82,83], and biases in visual information processing [83], all of which are commonly observed in depression. These findings are also consistent with the “atypical neurodevelopment” model, whereby reduced structural similarity in sensory cortices may weaken the early integration of external information and thereby increase vulnerability to depression [84,85]. Similarly, in the FEDN MDD subgroup, between-group differences in regional contributions to PBSI were also widely distributed, indicating that structural heterogeneity is already present at an early stage of the disorder.

### 4.4. Limitations

Several limitations of the present study should be acknowledged. First, the clinical complexity of MDD together with the differences in patient populations, scanners, and study protocols across sites may have biased our results. Here, appropriate and specific methodologies (e.g., ComBat harmonization) were adopted to rule out the possible effects of these confounding factors. Second, although age, sex, and education were statistically controlled in the present analyses, residual effects of demographic differences between patients with MDD and healthy controls cannot be fully excluded. Future studies with more closely matched samples are needed to further clarify the potential influence of demographic factors on PBSI alterations in MDD. Third, the cross-sectional and retrospective design limits the ability to infer causality regarding the link between altered individual-level structural similarity and MDD pathophysiology. Future prospective longitudinal designs are warranted to clarify these associations and to assess whether PBSI features can predict disease progression or treatment response using machine-learning models. Fourth, the episode status and medication history of many MDD patients were not clearly documented, which may have influenced our main findings. Although we conducted a subgroup analysis on the FEDN MDD patients, the markedly smaller sample size compared to the overall dataset limits the robustness and generalizability of our findings. Future neuroimaging studies based on larger and better-characterized FEDN MDD cohorts are warranted to provide more reliable and less biased evidence regarding the neural basis of MDD.

## 5. Conclusions

In conclusion, this large-scale, multi-site study applied the PBSI framework to characterize individual-level brain structural similarity in MDD across multiple cortical morphometric measures. Compared with healthy controls, patients with MDD showed lower PBSI across several morphometric metrics, indicating greater heterogeneity in cortical structural profiles. Furthermore, between-group differences in regional contributions to PBSI were widely distributed across both higher-order and lower-order large-scale networks. Similar patterns in the FEDN MDD subgroup further suggest that these alterations may already be detectable early in the course of the disorder. Overall, these findings support the utility of PBSI in capturing individualized structural variation in MDD and provide new evidence for the marked neurobiological heterogeneity of the disorder. They also extend current understanding of cortical structural “de-typicalization” in depression and suggest that PBSI may serve as a promising research-level neuroimaging marker for informing future studies on clinically meaningful subtypes and individualized intervention strategies.

## Data Availability

The original data presented in the study are openly available in: https://rfmri.org/REST-meta-MDD.

## Supplementary Materials

The following supporting information can be downloaded at: https://www.mdpi.com/article/10.3390/diagnostics16111632/s1, Figure S1: Number of regions showing significant between-group differences in PBSI regional contributions mapped onto Yeo 7-network; Tables S1–S6: Demographic, clinical, and statistical results; Supplementary File S1: Brain regions with significant between-group differences in PBSI contributions.

## References

[1] Malhi G.S., Mann J.J. (2018). Depression. Lancet.

[2] Fried E.I. (2017). The 52 symptoms of major depression: Lack of content overlap among seven common depression scales. J. Affect. Disord..

[3] Schmaal L., Hibar D.P., Sämann P.G., Hall G.B., Baune B.T., Jahanshad N., Cheung J.W., van Erp T.G.M., Bos D., Ikram M.A. (2016). Cortical abnormalities in adults and adolescents with major depression based on brain scans from 20 cohorts worldwide in the ENIGMA Major Depressive Disorder Working Group. Mol. Psychiatry.

[4] Schmaal L., Pozzi E., Ho T.C., van Velzen L.S., Veer I.M., Opel N., Van Someren E.J.W., Han L.K.M., Aftanas L., Aleman A. (2020). ENIGMA MDD: Seven years of global neuroimaging studies of major depression through worldwide data sharing. Transl. Psychiatry.

[5] Lorenzetti V., Allen N.B., Fornito A., Yücel M. (2009). Structural brain abnormalities in major depressive disorder: A selective review of recent MRI studies. J. Affect. Disord..

[6] Insel T.R. (2017). Digital phenotyping: Technology for a new science of behavior. JAMA.

[7] Laumann T.O., Zorumski C.F., Dosenbach N.U.F. (2023). Precision Neuroimaging for Localization-Related Psychiatry. JAMA Psychiatry.

[8] Allen P., Baldwin H., Bartholomeusz C.F., Chee M.W.L., Chen X., Cooper R.E., de Haan L., Hamilton H.K., He Y., Hegelstad W.T.V. (2024). Normative Modeling of Brain Morphometry in Clinical High Risk for Psychosis. JAMA Psychiatry.

[9] Shao J., Qin J., Wang H., Sun Y., Zhang W., Wang X., Wang T., Xue L., Yao Z., Lu Q. (2024). Capturing the Individual Deviations From Normative Models of Brain Structure for Depression Diagnosis and Treatment. Biol. Psychiatry.

[10] Sun X.Y., Sun J.R., Lu X.W., Dong Q.L., Zhang L., Wang W.X., Liu J., Ma Q., Wang X.Q., Wei D.T. (2023). Mapping Neurophysiological Subtypes of Major Depressive Disorder Using Normative Models of the Functional Connectome. Biol. Psychiatry.

[11] Rutherford S., Kia S.M., Wolfers T., Fraza C., Zabihi M., Dinga R., Berthet P., Worker A., Verdi S., Ruhe H.G. (2022). The normative modeling framework for computational psychiatry. Nat. Protoc..

[12] Doucet G.E., Moser D.A., Rodrigue A., Bassett D.S., Glahn D.C., Frangou S. (2019). Person-Based Brain Morphometric Similarity is Heritable and Correlates with Biological Features. Cereb. Cortex.

[13] Doucet G.E., Glahn D.C., Frangou S. (2020). Person-based similarity in brain structure and functional connectivity in bipolar disorder. J. Affect. Disord..

[14] Antoniades M., Haas S.S., Modabbernia A., Bykowsky O., Frangou S., Borgwardt S., Schmidt A. (2021). Personalized Estimates of Brain Structural Variability in Individuals with Early Psychosis. Schizophr. Bull..

[15] Jo Y.T., Lee J., Kim S.M., Park H., Joo S.W. (2025). Structural brain variability in recent-onset and chronic schizophrenia: Evidence from person-based similarity index analysis. Acta Neuropsychiatr..

[16] Baldwin H., Radua J., Antoniades M., Haas S.S., Frangou S., Agartz I., Allen P., Andreassen O.A., Atkinson K., Bachman P. (2022). Neuroanatomical heterogeneity and homogeneity in individuals at clinical high risk for psychosis. Transl. Psychiatry.

[17] Janssen J., Díaz-Caneja C.M., Alloza C., Schippers A., de Hoyos L., Santonja J., Gordaliza P.M., Buimer E.E.L., van Haren N.E.M., Cahn W. (2021). Dissimilarity in Sulcal Width Patterns in the Cortex can be Used to Identify Patients with Schizophrenia with Extreme Deficits in Cognitive Performance. Schizophr. Bull..

[18] Sun J., Zhao W., Xie Y., Zhou F., Wu L., Li Y., Li H., Li Y., Zeng C., Han X. (2023). Personalized estimates of morphometric similarity in multiple sclerosis and neuromyelitis optica spectrum disorders. NeuroImage Clin..

[19] Xie Y., Sun J., Man W., Zhang Z., Zhang N. (2023). Personalized estimates of brain cortical structural variability in individuals with Autism spectrum disorder: The predictor of brain age and neurobiology relevance. Mol. Autism.

[20] Fang K., Wen B., Liu L., Tian Y., Yang H., Han S., Sun X., Niu L. (2025). Two subtypes of major depressive disorder are identified from individualized gray matter morphological abnormalities in a large multi-site dataset. Psychol. Med..

[21] Yao C., Xiao Y., Wang P., Zheng Y., Sun J., Wang J., Xue S.W. (2025). Altered inter-subject variability of morphological brain networks and its association with neurotransmitters and gene expression in major depression disorder. J. Affect. Disord..

[22] Goya-Maldonado R., Erwin-Grabner T., Zeng L.L., Ching C.R.K., Aleman A., Amod A.R., Basgoze Z., Benedetti F., Besteher B., Brosch K. (2026). Classification of major depressive disorder using vertex-wise brain sulcal depth, curvature, and thickness with a deep and a shallow learning model. Mol. Psychiatry.

[23] Tamnes C.K., Østby Y., Fjell A.M., Westlye L.T., Due-Tønnessen P., Walhovd K.B. (2010). Brain Maturation in Adolescence and Young Adulthood: Regional Age-Related Changes in Cortical Thickness and White Matter Volume and Microstructure. Cereb. Cortex.

[24] Kong X.-Z., Mathias S.R., Guadalupe T., Glahn D.C., Franke B., Crivello F., Tzourio-Mazoyer N., Fisher S.E., Thompson P.M., Francks C. (2018). Mapping cortical brain asymmetry in 17,141 healthy individuals worldwide via the ENIGMA Consortium. Proc. Natl. Acad. Sci. USA.

[25] Winkler A.M., Kochunov P., Blangero J., Almasy L., Zilles K., Fox P.T., Duggirala R., Glahn D.C. (2010). Cortical thickness or grey matter volume? The importance of selecting the phenotype for imaging genetics studies. NeuroImage.

[26] Chen C.-H., Fiecas M., Gutiérrez E.D., Panizzon M.S., Eyler L.T., Vuoksimaa E., Thompson W.K., Fennema-Notestine C., Hagler D.J., Jernigan T.L. (2013). Genetic topography of brain morphology. Proc. Natl. Acad. Sci. USA.

[27] Keller S.S., Highley J.R., Garcia-Finana M., Sluming V., Rezaie R., Roberts N. (2007). Sulcal variability, stereological measurement and asymmetry of Broca’s area on MR images. J. Anat..

[28] Shin S.-J., Kim A., Han K.-M., Tae W.-S., Ham B.-J. (2022). Reduced Sulcal Depth in Central Sulcus of Major Depressive Disorder. Exp. Neurobiol..

[29] Arnone D., McIntosh A.M., Ebmeier K.P., Munafò M.R., Anderson I.M. (2012). Magnetic resonance imaging studies in unipolar depression: Systematic review and meta-regression analyses. Eur. Neuropsychopharmacol..

[30] Zhang B., Wu B., Zhang X., Xie H., Ling Y., Zhao Z., Gan R., Qiu L., Mechelli A., Jia Z. (2025). Gray matter structural alterations in first-episode drug-naïve adolescents with major depressive disorder: A comprehensive morphological analysis study. Psychol. Med..

[31] Hu S., Zhu L., Zhang X. (2025). Resolving heterogeneity in first-episode and drug-naive major depressive disorder based on individualized structural covariance network: Evidence from the REST-meta-MDD consortium. Psychol. Med..

[32] Luo Z., Hu Z., Qiu X., Li W., Wang C., Lan X., Mai S., Chen Y., Liu G., Zhang F. (2025). Resolving heterogeneity of early-onset major depressive disorder through individual differential structural covariance network analysis. J. Affect. Disord..

[33] Chen H., Liu P., Chen X., Liu J., Tang H., Tian Y., Wang X., Lu F., Zhou J. (2024). The mediation role of gray matter volume in the relationship between childhood maltreatment and psychological resilience in adolescents with first-episode major depressive disorder. Transl. Psychiatry.

[34] Gong Q., He Y. (2015). Depression, Neuroimaging and Connectomics: A Selective Overview. Biol. Psychiatry.

[35] Chen X., Lu B., Li H.-X., Li X.-Y., Wang Y.-W., Castellanos F.X., Cao L.-P., Chen N.-X., Chen W., Cheng Y.-Q. (2022). The DIRECT consortium and the REST-meta-MDD project: Towards neuroimaging biomarkers of major depressive disorder. Psychoradiology.

[36] Monereo-Sánchez J., de Jong J.J.A., Drenthen G.S., Beran M., Backes W.H., Stehouwer C.D.A., Schram M.T., Linden D.E.J., Jansen J.F.A. (2021). Quality control strategies for brain MRI segmentation and parcellation: Practical approaches and recommendations—Insights from the Maastricht study. NeuroImage.

[37] Yan C.-G., Wang X.-D., Lu B. (2021). DPABISurf: Data processing & analysis for brain imaging on surface. Sci. Bull..

[38] Dale A.M., Fischl B., Sereno M.I. (1999). Cortical surface-based analysis—I. Segmentation and surface reconstruction. Neuroimage.

[39] Avants B.B., Tustison N., Song G. (2009). Advanced normalization tools (ANTS). Insight J..

[40] Jenkinson M., Bannister P., Brady M., Smith S. (2002). Improved Optimization for the Robust and Accurate Linear Registration and Motion Correction of Brain Images. NeuroImage.

[41] Ashburner J. (2012). SPM: A history. NeuroImage.

[42] Winkler A.M., Ridgway G.R., Webster M.A., Smith S.M., Nichols T.E. (2014). Permutation inference for the general linear model. NeuroImage.

[43] Li X., Morgan P.S., Ashburner J., Smith J., Rorden C. (2016). The first step for neuroimaging data analysis: DICOM to NIfTI conversion. J. Neurosci. Methods.

[44] Tange O. (2011). GNU Parallel: The Command-Line Power Tool. Usenix Mag..

[45] Gorgolewski K.J., Auer T., Calhoun V.D., Craddock R.C., Das S., Duff E.P., Flandin G., Ghosh S.S., Glatard T., Halchenko Y.O. (2016). The brain imaging data structure, a format for organizing and describing outputs of neuroimaging experiments. Sci. Data.

[46] Tustison N.J., Avants B.B., Cook P.A., Yuanjie Z., Egan A., Yushkevich P.A., Gee J.C. (2010). N4ITK: Improved N3 Bias Correction. IEEE Trans. Med. Imaging.

[47] Ségonne F., Dale A.M., Busa E., Glessner M., Salat D., Hahn H.K., Fischl B. (2004). A hybrid approach to the skull stripping problem in MRI. NeuroImage.

[48] Doucet G.E., Lin D., Du Y., Fu Z., Glahn D.C., Calhoun V.D., Turner J., Frangou S. (2020). Personalized estimates of morphometric similarity in bipolar disorder and schizophrenia. npj Schizophr..

[49] Iseli G.C., Ulrich S., Stämpfli P., Studerus E., Coynel D., Riecher-Rössler A., Homan P., Kaiser S., Borgwardt S., Kirschner M. (2024). Parsing heterogeneity in global and local white matter integrity at different stages across the psychosis continuum. Schizophrenia.

[50] Joo S.W., Jo Y.T., Kim Y., Lee W.H., Chung Y.C., Lee J.S. (2024). Structural variability of the cerebral cortex in schizophrenia and its association with clinical symptoms. Psychol. Med..

[51] Omlor W., Rabe F., Fuchs S., Surbeck W., Cecere G., Huang G.-Y., Homan S., Kallen N., Georgiadis F., Spiller T. (2025). Estimating Multimodal Structural Brain Variability in Schizophrenia Spectrum Disorders: A Worldwide ENIGMA Study. Am. J. Psychiatry.

[52] Andrews-Hanna J.R., Smallwood J., Spreng R.N. (2014). The default network and self-generated thought: Component processes, dynamic control, and clinical relevance. Ann. N. Y. Acad. Sci..

[53] Fortin J.-P., Cullen N., Sheline Y.I., Taylor W.D., Aselcioglu I., Cook P.A., Adams P., Cooper C., Fava M., McGrath P.J. (2018). Harmonization of cortical thickness measurements across scanners and sites. NeuroImage.

[54] Duman R.S., Aghajanian G.K., Sanacora G., Krystal J.H. (2016). Synaptic plasticity and depression: New insights from stress and rapid-acting antidepressants. Nat. Med..

[55] Li J., Seidlitz J., Suckling J., Fan F., Ji G.-J., Meng Y., Yang S., Wang K., Qiu J., Chen H. (2021). Cortical structural differences in major depressive disorder correlate with cell type-specific transcriptional signatures. Nat. Commun..

[56] Bethlehem R.A.I., Seidlitz J., White S.R., Vogel J.W., Anderson K.M., Adamson C., Adler S., Alexopoulos G.S., Anagnostou E., Areces-Gonzalez A. (2022). Brain charts for the human lifespan. Nature.

[57] Marquand A.F., Kia S.M., Zabihi M., Wolfers T., Buitelaar J.K., Beckmann C.F. (2019). Conceptualizing mental disorders as deviations from normative functioning. Mol. Psychiatry.

[58] Verdi S., Marquand A.F., Schott J.M., Cole J.H. (2021). Beyond the average patient: How neuroimaging models can address heterogeneity in dementia. Brain.

[59] Wu B., Chen Y., Long X., Cao Y., Xie H., Wang X., Roberts N., Gong Q., Jia Z. (2023). Altered single-subject gray matter structural networks in first-episode drug-naïve adolescent major depressive disorder. Psychiatry Res..

[60] Shen A., Shi K., Xia Q., Gong W., Huang Y., Wang Y., Zhai Q., Yan R., Yao Z., Lu Q. (2025). Surface-based analysis of early cortical gyrification and thickness alterations in treatment-Naïve, first-episode depressive patients during emerging adulthood. J. Affect. Disord..

[61] Wohleb E.S., Terwilliger R., Duman C.H., Duman R.S. (2018). Stress-Induced Neuronal Colony Stimulating Factor 1 Provokes Microglia-Mediated Neuronal Remodeling and Depressive-like Behavior. Biol. Psychiatry.

[62] Sheline Y.I., Sanghavi M., Mintun M.A., Gado M.H. (1999). Depression Duration But Not Age Predicts Hippocampal Volume Loss in Medically Healthy Women with Recurrent Major Depression. J. Neurosci..

[63] Kempton M.J., Salvador Z., Munafò M.R., Geddes J.R., Simmons A., Frangou S., Williams S.C.R. (2011). Structural Neuroimaging Studies in Major Depressive Disorder. Arch. Gen. Psychiatry.

[64] Sun B.B., Loomis S.J., Pizzagalli F., Shatokhina N., Painter J.N., Foley C.N., Sun B., Tsai E., Bronson P., Sexton D. (2022). Genetic map of regional sulcal morphology in the human brain from UK biobank data. Nat. Commun..

[65] van der Meer D., Kaufmann T., Shadrin A.A., Makowski C., Frei O., Roelfs D., Monereo-Sánchez J., Linden D.E.J., Rokicki J., Alnæs D. (2021). The genetic architecture of human cortical folding. Sci. Adv..

[66] Van Essen D.C. (2020). A 2020 view of tension-based cortical morphogenesis. Proc. Natl. Acad. Sci. USA.

[67] Solhtalab A., Foroughi A.H., Pierotich L., Razavi M.J. (2025). Stress landscape of folding brain serves as a map for axonal pathfinding. Nat. Commun..

[68] Grasby K.L., Jahanshad N., Painter J.N., Colodro-Conde L., Bralten J., Hibar D.P., Lind P.A., Pizzagalli F., Ching C.R.K., McMahon M.A.B. (2020). The genetic architecture of the human cerebral cortex. Science.

[69] Storsve A.B., Fjell A.M., Tamnes C.K., Westlye L.T., Overbye K., Aasland H.W., Walhovd K.B. (2014). Differential Longitudinal Changes in Cortical Thickness, Surface Area and Volume across the Adult Life Span: Regions of Accelerating and Decelerating Change. J. Neurosci..

[70] Sheline Y.I., Barch D.M., Price J.L., Rundle M.M., Vaishnavi S.N., Snyder A.Z., Mintun M.A., Wang S., Coalson R.S., Raichle M.E. (2009). The default mode network and self-referential processes in depression. Proc. Natl. Acad. Sci. USA.

[71] Zhou H.-X., Chen X., Shen Y.-Q., Li L., Chen N.-X., Zhu Z.-C., Castellanos F.X., Yan C.-G. (2020). Rumination and the default mode network: Meta-analysis of brain imaging studies and implications for depression. NeuroImage.

[72] Menon V. (2011). Large-scale brain networks and psychopathology: A unifying triple network model. Trends Cogn. Sci..

[73] Menon V., Uddin L.Q. (2010). Saliency, switching, attention and control: A network model of insula function. Brain Struct. Funct..

[74] Kaiser R.H., Andrews-Hanna J.R., Wager T.D., Pizzagalli D.A. (2015). Large-Scale Network Dysfunction in Major Depressive Disorder: A Meta-analysis of Resting-State Functional Connectivity. JAMA Psychiatry.

[75] Han Y., Gao Y., Wang S., Lin X., Li P., Liu W., Lu L., Wang C. (2025). Cortical folding in distinguishing first-episode bipolar and unipolar depression. J. Affect. Disord..

[76] Kang Y., Kang W., Kim A., Tae W.-S., Ham B.-J., Han K.-M. (2023). Decreased cortical gyrification in major depressive disorder. Psychol. Med..

[77] Lui S., Wu Q., Qiu L., Yang X., Kuang W., Chan R.C., Huang X., Kemp G.J., Mechelli A., Gong Q. (2011). Resting-state functional connectivity in treatment-resistant depression. Am. J. Psychiatry.

[78] Wüthrich F., Lefebvre S., Mittal V.A., Shankman S.A., Alexander N., Brosch K., Flinkenflügel K., Goltermann J., Grotegerd D., Hahn T. (2023). The neural signature of psychomotor disturbance in depression. Mol. Psychiatry.

[79] Song X.M., Liu D.Y., Hirjak D., Hu X.W., Han J.F., Roe A.W., Yao D.Z., Tan Z.L., Northoff G. (2024). Motor versus Psychomotor? Deciphering the Neural Source of Psychomotor Retardation in Depression. Adv. Sci..

[80] de Erausquin G.A., Bracht T., Federspiel A., Schnell S., Horn H., Höfle O., Wiest R., Dierks T., Strik W., Müller T.J. (2012). Cortico-Cortical White Matter Motor Pathway Microstructure Is Related to Psychomotor Retardation in Major Depressive Disorder. PLoS ONE.

[81] Kairys A.E., Schmidt-Wilcke T., Puiu T., Ichesco E., Labus J.S., Martucci K., Farmer M.A., Ness T.J., Deutsch G., Mayer E.A. (2015). Increased Brain Gray Matter in the Primary Somatosensory Cortex is Associated with Increased Pain and Mood Disturbance in Patients with Interstitial Cystitis/Painful Bladder Syndrome. J. Urol..

[82] Surguladze S., Brammer M.J., Keedwell P., Giampietro V., Young A.W., Travis M.J., Williams S.C.R., Phillips M.L. (2005). A differential pattern of neural response toward sad versus happy facial expressions in major depressive disorder. Biol. Psychiatry.

[83] Wu F., Lu Q., Kong Y., Zhang Z. (2023). A Comprehensive Overview of the Role of Visual Cortex Malfunction in Depressive Disorders: Opportunities and Challenges. Neurosci. Bull..

[84] He Y., Peng D., Shi F., Li G., Fralick D., Shen T., Qiu M., Liu J., Jiang K., Shen D. (2015). Surface Vulnerability of Cerebral Cortex to Major Depressive Disorder. PLoS ONE.

[85] Bick J., Nelson C.A. (2015). Early Adverse Experiences and the Developing Brain. Neuropsychopharmacology.

