# Supplementary material for: Personalized Estimates of Brain Cortical Structural Similarity in Major Depressive Disorder: Evidence from a Multi-Site Neuroimaging Dataset
Source: Diagnostics (Basel). 2026 May 26;16(11):1632. doi: 10.3390/diagnostics16111632 (PMC13257425; doi:10.3390/diagnostics16111632)
Supplement: Supplementary file 1 [file diagnostics-16-01632-s001.zip › diagnostics-4312230-supplementary/Supplementary Tables and Figure.pdf]

Supplementary Materials

Table S1. Demographic and clinical characteristics of the samples across 22 sites.

	MDD patients											Healthy controls			
Site	Number	Age (years)	Sex (M/F)	Education (years)	Onset age (years)	Illness duration (months)	First-episode (yes/no/NA)	Episode number	Medication (yes/no/NA)	HAMD	HAMA	Number	Age (years)	Sex (M/F)	Education (years)
1	262	38.54±12.12 (18-65)	93/169	12.06±3.65 (0-18)	35.66±12.43 (11-65)	50.15±65.21 (1-360)	198/46/18	1.19±0.40 (1-3)	119/117/26	21.00±5.69 (0-35)	22.70±9.11 (0-47)	251	39.64±15.87 (19-64)	87/164	12.98±3.90 (1-20)
2	78	21.49±3.05 (18-30)	26/52	13.33±1.85 (9-17)	18.88±4.81 (8-28)	18.38±27.14 (0-120)	74/4/0	1.09±0.43 (1-4)	9/25/44	17.88±9.67 (0-52)	16.56±5.48 (8-28)	59	21.15±2.72 (18-32)	23/36	13.85±1.72 (12-16)
3	20	34.40±10.56 (20-60)	5/15	14.90±2.97 (9-18)	35.55±11.96 (23-59)	14.50±29.14 (1-96)	18/2/0	1.15±0.49 (1-3)	0/20/0	29.37±6.41 (18-44)	20.74±9.12 (6-38)	20	26.50±11.22 (19-58)	5/15	15.40±1.47 (12-18)
4	32	32.25±10.38 (19-51)	14/18	12.53±4.29 (1-19)	28.19±11.22 (13-50)	42.45±57.46 (2-216)	15/15/2	2.50±3.60 (1-20)	13/18/1	21.76±5.72 (0-32)	-	28	30.14±9.79 (18-49)	10/18	13.00±3.73 (6-20)
5	132	33.70±9.98 (18-53)	59/73	12.89±3.45 (6-18)	-	-	132/0/0	1	0/0/132	26.48±3.74 (19-39)	15.08±4.27 (8-28)	60	37.10±8.96 (20-61)	33/27	14.33±2.68 (9-18)
6	65	31.37±9.14 (18-51)	35/30	12.11±3.06 (6-19)	28.50±9.36 (13-48)	44.61±60.77 (0-360)	24/36/5	1.89±1.13 (1-6)	55/10/0	20.46±6.43 (6-40)	16.12±9.04 (3-41)	105	23.60±5.11 (18-42)	46/59	13.90±2.27 (9-19)
7	13	23.38±5.97 (18-33)	2/11	11.85±2.82 (9-16)	22.54±6.67 (14-33)	11.52±16.67 (0-48)	12/1/0	1.08±0.28 (1-2)	10/3/0	27.62±12.36 (6-50)	13.38±9.55 (4-36)	22	21.45±3.00 (18-28)	8/14	12.73±2.25 (9-16)
8	38	31.84±8.99 (18-55)	15/23	15.76±1.93 (9-22)	29.58±8.79 (16-51)	21.63±11.06 (3-57)	36/2/0	3.68±2.41 (1-10)	2/36/0	24.42±4.02 (17-32)	18.50±4.69 (13-27)	33	32.85±9.38 (21-56)	15/18	15.36±2.00 (9-18)
9	43	46.65±12.30 (21-64)	17/26	10.93±4.70 (0-21)	43.81±12.61 (18-63)	35.10±53.93 (0-240)	30/13/0	1.42±0.76 (1-4)	43/0/0	-	-	26	36.69±13.88 (19-64)	10/16	13.46±3.96 (5-19)
10	84	34.70±12.44 (18-65)	37/47	13.19±3.02 (6-16)	26.78±11.15 (6-58)	93.11±96.14 (1-480)	31/36/17	2.39±1.87 (0-10)	62/15/7	14.58±8.06 (0-35)	14.56±8.60 (0-40)	69	36.38±12.56 (19-60)	31/38	14.62±2.28 (9-16)
11	111	39.35±12.34 (18-61)	53/58	10.97±3.51 (4-19)	32.89±12.18 (11-58)	80.05±97.69 (0-432)	33/74/4	3.57±4.00 (1-30)	108/0/3	23.92±9.06 (1-49)	-	68	36.62±12.11 (20-59)	30/38	12.96±3.57 (3-18)
12	63	30.51±7.15 (18-45)	21/42	13.75±3.36 (9-21)	29.68±7.06 (18-44)	6.05±4.20 (1-12)	63/0/0	1	0/63/0	21.32±3.46 (15-32)	-	32	29.59±5.00 (21-44)	15/17	14.59±2.82 (9-19)
13	33	38.36±14.48 (18-60)	14/19	12.15±3.87 (0-18)	32.88±13.77 (15-60)	-	8/25/0	1.45±1.12 (0-4)	0/0/33	18.36±9.66 (0-35)	11.58±5.76 (3-26)	29	35.45±13.76 (22-59)	9/20	11.28±4.31 (0-16)
14	139	43.83±11.40 (18-64)	48/97	9.02±3.60 (0-16)	37.83±11.56 (12-62)	67.52±76.85 (0-339)	42/97/0	2.53±2.13 (1-21)	139/0/0	29.88±10.54 (2-58)	-	144	38.25±12.08 (18-60)	66/78	13.21±4.25 (4-21)
15	28	23.14±4.44 (18-33)	3/25	12.32±2.31 (9-19)	23.14±4.44 (18-33)	15.32±17.20 (2-72)	20/8/0	2.29±2.63 (1-8)	0/28/0	-	-	34	21.65±4.58 (18-35)	23/11	12.59±1.44 (12-16)
16	80	26.41±7.59 (18-51)	35/45	13.91±2.59 (8-19)	23.32±8.11 (8-49)	30.68±44.91 (0-180)	44/36/0	1.75±1.12 (1-6)	1/79/0	25.53±3.78 (20-38)	13.35±7.66 (4-40)	81	25.73±7.38 (18-52)	44/37	15.53±2.81 (7-22)
17	46	35.91±9.98 (20-56)	18/28	12.43±4.03 (3-20)	35.61±10.07 (20-56)	4.11±2.85 (0-12)	46/0/0	1	0/46/0	23.48±5.02 (18-38)	22.17±5.73 (11-41)	50	34.68±9.56 (19-54)	19/31	14.12±4.37 (5-20)
18	34	35.26±12.64 (18-60)	18/16	12.74±4.63 (0-16)	-	19.61±21.51 (0-67)	15/19/0	1.68±0.91 (1-4)	0/34/0	22.29±4.43 (18-34)	-	34	32.74±11.78 (18-57)	14/20	12.15±3.32 (6-16)
19	30	27.70±11.09	5/25	12.90±3.17	26.22±10.93	13.30±14.77	21/9/0	1.30±0.47	15/15/0	22.80±5.60	20.93±6.38	32	30.94±7.32	16/16	13.94±2.84

		(18-52)		(6-17)	(16-51)	(0-48)		(1-2)		(11-34)	(9-35)		(21-46)		(9-19)
20	16	30.69±7.60 (22-51)	4/12	14.94±3.79 (9-26)	29.25±8.43 (17-51)	17.50±15.57 (1-54)	10/6/0	1.38±0.50 (1-2)	2/14/0	25.31±7.16 (11-36)	-	20	31.40±10.99 (23-59)	5/15	16.10±3.21 (9-20)
21	60	32.98±11.09 (18-61)	15/45	12.33±4.17 (0-22)	32.30±11.23 (17-59)	35.56±45.45 (1-240)	19/16/25	-	0/1/59	22.86±2.55 (18-27)	17.33±6.20 (9-31)	56	32.80±11.06 (19-61)	31/25	15.88±4.59 (5-25)
22	35	35.74±10.02 (19-53)	10/25	13.20±3.75 (0-18)	31.23±7.91 (19-45)	5.59±14.93 (1-72)	20/15/0	1.06±1.35 (0-4)	16/19/0	22.29±6.04 (14-36)	21.31±7.54 (6-38)	24	33.67±11.82 (18-56)	8/16	14.46±2.32 (9-18)

Data are expressed as mean ± standard deviation. Numbers in parentheses are the range. Abbreviations: F, female; HAMA, Hamilton Rating Scale for Anxiety; HAMD, Hamilton Rating Scale for Depression; M, male; MDD, major depressive disorder; NA, not available. Onset age is missing for 304 patients. Illness duration is missing for 293 patients. First-episode information is missing for 71 patients. Episode number is missing for 146 patients. Medication information is missing for 312 patients. HAMD is missing for 173 patients. HAMA is missing for 644 patients.

Table S2. Statistical characteristics of all participants.

Characteristic	MDD patients	Healthy controls	Statistic	*P* value
Number	1442	1277	-	-
Age (years)	34.83±12.17 (18-65)	33.05±12.78 (18-64)	$t = 3.71$	< 0.001
Sex (M/F)	547/895	548/729	$\chi^2 = 6.78$	< 0.05
Education (years)	12.31±3.73 (0-26)	13.78±3.51 (0-25)	$t = -10.58$	< 0.001
Onset age (years)	31.76±12.02 (6-65)	-	-	-
Illness duration (months)	44.42±66.82 (0-480)	-	-	-
First-episode (yes/no/NA)	911/441/90	-	-	-
Episode number	1.74±1.85 (0-30)	-	-	-
Medication (yes/no/NA)	594/536/312	-	-	-
HAMD	22.82±8.03 (0-58)	-	-	-
HAMA	18.35±8.54 (0-47)	-	-	-

Data are expressed as mean ± standard deviation. Numbers in parentheses are the range. Abbreviations: F, female; HAMA, Hamilton Rating Scale for Anxiety; HAMD, Hamilton Rating Scale for Depression; M, male; MDD, major depressive disorder; NA, not available.

Table S3. Demographic and clinical characteristics of patients with first-episode drug-naive MDD.

Patients with first-episode drug-naive MDD											
Site	Number	Age (years)	Sex (M/F)	Education (years)	Onset age (years)	Illness duration (months)	First-episode (yes/no/NA)	Episode number	Medication (yes/no/NA)	HAMD	HAMA
1	55	37.96±11.76 (20-64)	15/40	12.34±4.1 (0-18)	38±11.87 (20-64)	5.45±4.42 (1-12)	55/0/0	1	0/55/0	22.80±4.36 (15-33)	23.87±7.86 (8-45)
2	14	20.86±3.48 (18-28)	6/8	12.5±2.21 (9-16)	20.5±3.76 (17-28)	4.93±3.69 (1-12)	14/0/0	1	0/14/0	15.71±9.86 (0-30)	-
3	8	37.5±12.58 (23-60)	2/6	14.25±3.73 (9-18)	37.38±12.33 (23-59)	3.88±3.44 (1-12)	8/0/0	1	0/8/0	27.75±6.78 (18-39)	16.75±10.01 (6-35)
4	4	24.75±2.36 (23-28)	3/1	13.75±3.2 (9-16)	24.75±3.10 (22-29)	3.75±2.22 (2-7)	4/0/0	1	0/4/0	23.00±4.69 (18-27)	-
6	4	27±4.55 (22-33)	2/2	14.5±3.70 (9-17)	27±4.24 (23-33)	3.13±1.93 (2-6)	4/0/0	1	0/4/0	19±6.58 (11-27)	12.75±2.36 (11-16)
7	2	18.00±0.00 (18-18)	1/1	10.50±2.12 (9-12)	18.00±0.00 (18-18)	3.50±3.54 (1-6)	2/0/0	1	0/2/0	23.00±24.04 (6-40)	12.00±11.31 (4-20)
8	2	27.50±7.78 (22-33)	1/1	16.00±0 (16-16)	26.50±9.19 (20-33)	6.50±0.71 (6-7)	2/0/0	1	0/2/0	20.50±2.12 (19-22)	-
12	63	30.51±7.15 (18-45)	21/42	13.75±3.36 (9-21)	29.68±7.06 (18-44)	6.05±4.20 (1-12)	63/0/0	1	0/63/0	21.32±3.46 (15-32)	-
16	30	24.6±6.23 (18-44)	12/18	13.77±2.85 (8-19)	24.17±6.37 (18-44)	4.59±3.66 (1-12)	30/0/0	1	0/30/0	25.40±3.02 (20-30)	13.27±7.55 (4-33)
17	46	35.91±9.98 (20-56)	18/28	12.43±4.03 (3-20)	35.61±10.07 (20-56)	4.11±2.85 (0-12)	46/0/0	1	0/46/0	23.48±5.02 (18-38)	22.17±5.73 (11-41)
19	10	21.2±2.1 (18-25)	4/6	14.2±2.44 (9-17)	20.85±2.29 (17-25)	5.8±4.57 (1-12)	10/0/0	1	0/10/0	22.9±4.84 (13-30)	22.1±7.16 (10-32)
20	5	37.4±9.7 (28-51)	3/2	12.6±3.51 (9-16)	37.2±9.83 (28-51)	5.6±4.04 (1-12)	5/0/0	1	0/5/0	25.8±5.36 (21-33)	-

Data are expressed as mean ± standard deviation. Numbers in parentheses are the range. Abbreviations: F, female; HAMA, Hamilton Rating Scale for Anxiety; HAMD, Hamilton Rating Scale for Depression; M, male; MDD, major depressive disorder; NA, not available. HAMA is missing for 102 patients.

Table S4. Statistical characteristics of patients with first-episode drug-naive MDD and healthy controls.

Characteristic	Patients with first-episode drug-naive MDD	Healthy controls	Statistic	*P* value
Number	243	1277	-	-
Age (years)	31.64±10.47 (18-64)	33.05±12.78 (18-64)	t = -1.85	0.065
Sex (M/F)	88/155	548/729	χ^2 = 3.49	0.062
Education (years)	13.13±3.56 (0-21)	13.78±3.51 (0-25)	t = -2.61	< 0.05
Onset age (years)	31.09±10.52 (17-64)	-	-	-
Illness duration (months)	5.11±3.87 (0-12)	-	-	-
First-episode (yes/no/NA)	243/0/0	-	-	-
Episode number	1	-	-	-
Medication (yes/no/NA)	0/243/0	-	-	-
HAMD	22.42±5.54 (0-40)	-	-	-
HAMA	21.14±8.02 (4-45)	-	-	-

Data are expressed as mean ± standard deviation. Numbers in parentheses are the range. Abbreviations: F, female; HAMA, Hamilton Rating Scale for Anxiety; HAMD, Hamilton Rating Scale for Depression; M, male; MDD, major depressive disorder; NA, not available.

Table S5. Comparison of PBSI scores between MDD patients and healthy controls.

Metric	CT	CV	SA	SD
β	−0.00488	−0.00398	0.00264	−0.00072
95% CI	[−0.00675, −0.00302]	[−0.00553, −0.00244]	[−0.00118, 0.00646]	[−0.00117, −0.00026]
t	−5.13	−5.05	1.35	−3.09
P	<0.001	<0.001	0.176	0.002
Cohen's d	−0.24	−0.23	0.05	−0.14

Abbreviations: PBSI, person-based similarity index; MDD, major depressive disorder; HC, healthy control; CT, cortical thickness; CV, cortical volume; SA, surface area; SD, sulcal depth.

Table S6. Comparison of PBSI scores between FEDN MDD patients and healthy controls.

Metric	CT	CV	SA	SD
β	−0.00045	−0.00347	−0.01288	−0.00167
95% CI	[−0.00375, 0.00284]	[−0.00616, −0.00077]	[−0.01957, −0.00619]	[−0.00246, −0.00088]
t	−0.27	−2.52	−3.78	−4.13
P	0.788	0.012	<0.001	<0.001
Cohen's d	0.07	−0.11	−0.29	−0.28

Abbreviations: PBSI, person-based similarity index; FEDN MDD, first-episode drug-naïve major depressive disorder; HC, healthy control; CT, cortical thickness; CV, cortical volume; SA, surface area; SD, sulcal depth.

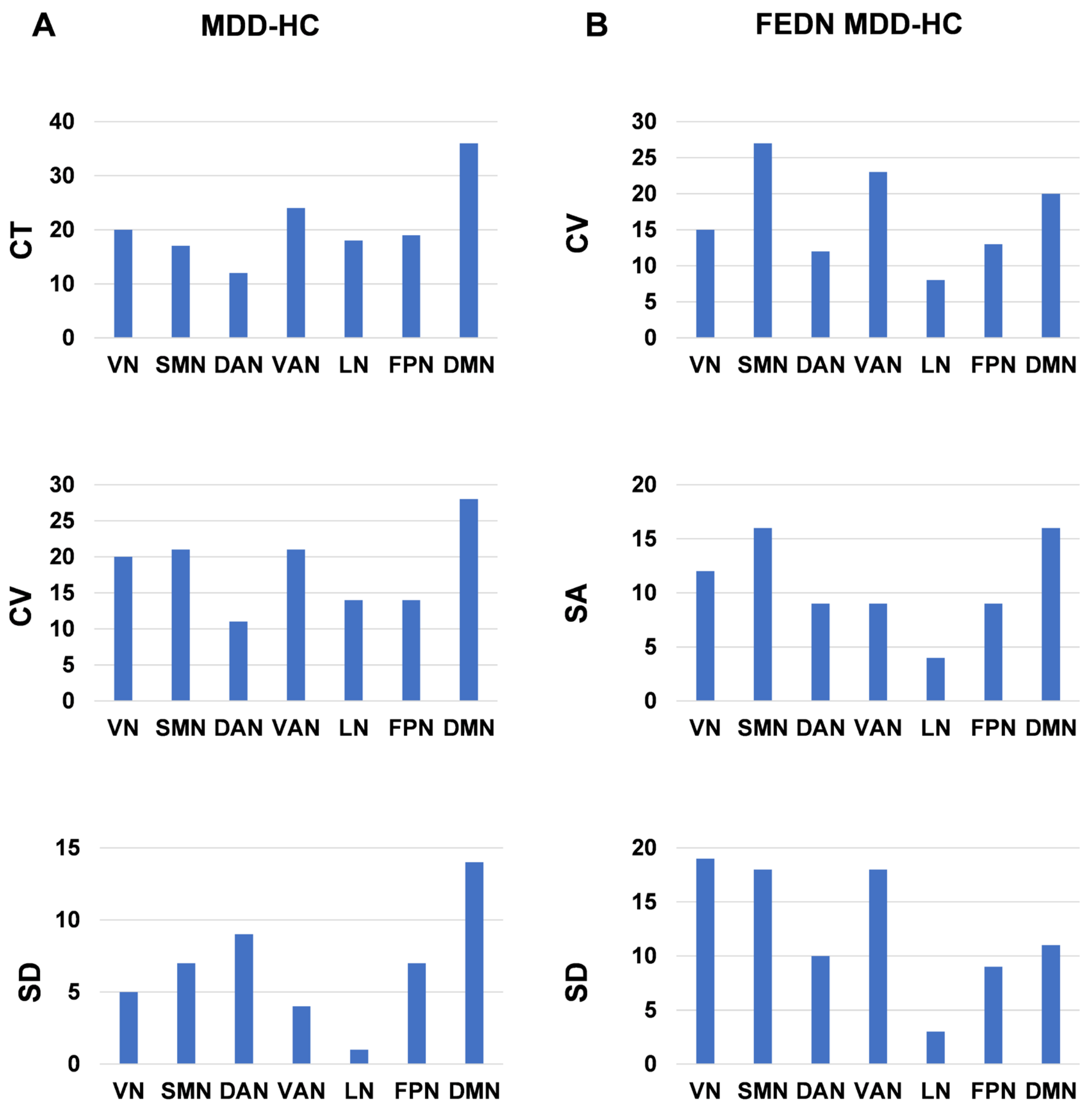

Figure S1. Number of regions showing significant between-group differences in PBSI regional contributions mapped onto Yeo 7-network. Abbreviations: CT, cortical thickness; CV, cortical volume; DAN, dorsal attention network; DMN, default mode network; FEDN, first-episode drug-naive; FPN, frontoparietal network; LN, limbic network; MDD, major depressive disorder; SA, surface area; SD, sulcal depth; SMN, somatomotor network; VAN, ventral attention network; VN, visual network.
